# Changes in menstrual cycles among Iranian women during the COVID-19 pandemic: A cross-sectional study

**DOI:** 10.18502/ijrm.v22i4.16389

**Published:** 2024-06-12

**Authors:** Fahimeh Ranjbar, Shima Haghani, Motahareh Aghashahi, Maryam Gharacheh

**Affiliations:** ^1^Nursing and Midwifery Care Research Center, Health Management Research Institute, Iran University of Medical Sciences, Tehran, Iran.; ^2^School of Health Management and Information Sciences, Iran University of Medical Sciences, Tehran, Iran.

**Keywords:** Menstrual cycle, Menstrual irregularities, COVID-19, Vaccine.

## Abstract

**Background:**

Some women experienced alterations in their menstrual cycles during the Coronavirus disease 2019 (COVID-19) pandemic.

**Objective:**

This study aimed to evaluate the changes in menstrual cycles among Iranian women during the COVID-19 pandemic.

**Materials and Methods:**

This cross-sectional study included 1500 women of childbearing age referred to healthcare centers in Tehran, Iran from April-November 2022. Participants were selected using the stratified sampling method. Data were collected via a questionnaire covering information on menstrual cycle, COVID-19 infection, and COVID-19 vaccination status. The study's outcomes focused on menstrual changes by evaluating cycle regularity, duration, and symptoms based on participant-reported observations.

**Results:**

The findings revealed that the pattern of menstrual change during the pandemic occurred in the form of a shorter interval in 8.6%, and longer interval in 8.9%, shorter duration in 10.4%, longer duration in 9.4%, heavier bleeding in 12.3%, lighter bleeding in 14.7%. The chance of menstrual changes in women who were infected with COVID-19 was 54% higher than that of women who were not infected, and the chance of menstrual changes in women who received 3 doses of COVID-19 vaccine was 1.5 times higher than women who had not been vaccinated.

**Conclusion:**

Our research revealed a high incidence of menstrual changes among Iranian women of childbearing age during the COVID-19 pandemic. Healthcare providers should be knowledgeable about the menstrual changes associated with COVID-19 infection and vaccination to provide information and support to affected women.

## 1. Introduction

Research on menstrual irregularities in women during the Coronavirus disease 2019 (COVID-19) pandemic is limited and contradictory (1). In the US, more than half of the women reported changes in their menstrual cycle, including alterations in cycle length, duration of menses, and premenstrual symptom changes (2). Similarly, in Italy (3) and Turkey (4) women reported changes in their menstrual cycle during the pandemic compared to the pre-pandemic period. Conversely, a study in the US, focusing on women who used a cell phone app to track menstrual cycles, found no significant alteration in menstruation during the pandemic (5).

It has been reported that women experiencing high levels of perceived stress during the COVID-19 pandemic are more likely to have longer duration of menses and heavier bleeding compared to those with moderate perceived stress scores (6). This suggests that the elevated anxiety and stress levels associated with the COVID-19 pandemic can impact the characteristics of the menstrual cycle in women. Notably, a study conducted in Turkey revealed higher levels of anxiety were associated with increased menstrual symptoms, while simultaneously reducing the length of menses (7). Additionally, among female health workers, anxiety caused by the COVID-19 pandemic, perceived stress, and depressive symptoms were correlated with an increased prevalence of menstrual irregularities (8).

The findings of a study conducted in Spain suggested COVID-19 may have an impact on menstrual patterns. However, the evidence regarding this association was inconclusive, even though women with long-term COVID-19 appeared to be at greater risk of reporting menstrual abnormalities (9). Another study examined the average concentration of sex hormones and ovarian reserve in women of reproductive age who contracted COVID-19 and found no significant changes (10). While unexpected menstrual changes are not officially listed as side effects of a COVID-19 vaccine, there have been reports from physicians and health professionals regarding menstrual abnormalities shortly after vaccination. However, it is important to note that most women who experience a change in their menstrual cycle after vaccination find that it returns to normal in the following cycle (11). A UK study revealed that approximately 20% of women experienced menstrual irregularities after receiving the COVID-19 vaccine (12). A potential link was also found between the COVID-19 vaccine and menstrual abnormalities in Jordan (13). Furthermore, regardless of the type of vaccine administered, about 50–60% of women reported menstrual irregularities after receiving the first dose of the COVID-19 vaccine (14).

While several studies have indicated certain women may experience alterations in their menstrual cycle during the COVID-19 pandemic, potentially due to stress related to the pandemic, the virus itself, or the vaccination process, it is essential to conduct further research to explore the impact of COVID-19 on women's menstrual health (1, 15). Currently, there is limited research on menstrual health during the COVID-19 pandemic, specifically in Iran. A recent study conducted in Iran revealed a high prevalence of menstrual disturbances among women, irrespective of their vaccination status (16).

Considering the importance of menstrual health for women in developing countries, this study aimed to evaluate the changes in menstrual cycles among Iranian women during the COVID-19 pandemic.

## 2. Materials and Methods

This cross-sectional study was conducted on 1500 women of childbearing age between April and November 2022. The study targeted women of childbearing age (15–45 yr) who sought healthcare services at healthcare centers in Tehran, Iran. Pregnant and breastfeeding women within the past 2 yr, as well as women experiencing menopause (either natural or surgical) or pre-menopause, were excluded from the study. Additionally, women with active sexually transmitted diseases, cervicitis, endometriosis, and fibroids were excluded from the study.

### Sample size

The sample size for this study was determined using a formula for estimating the appropriate sample size for a single population proportion. Several assumptions were taken into consideration during the calculations. The prevalence of menstrual abnormalities (p = 19%) was based on previous research in Iran (17). A 95% confidence interval and a 5% margin of error were used, with a degree of precision set at 0.02. Based on these parameters, it was determined that a total sample size of 1480 participants was necessary for the study. 


$$n=\frac{z_{1-\propto /2}^{2}pq}{d^{2}}=\frac{1.96^{2}\times 0.19\times 0.81}{0.02^{2}}\approx 1480$$

### Data collection

Women were recruited for the study using the stratified sampling method. Initially, the urban public health centers affiliated with the Iran University of Medical Sciences, Tehran, Iran were divided into 2 subgroups based on their location: west and northwest. Within each subgroup, 2 health centers were randomly selected. A total of 1500 women were consecutively invited to participate in the study, and those who provided written consent were included in the survey. Women were recruited for the study at urban healthcare centers, where midwives screened them for eligibility. Eligible participants were provided with an explanation of the study's purpose, after which those who agreed to participate were given the questionnaire to self-administer.

### Instrument

Data collection involved the use of a demographic and reproductive characteristics checklist. This checklist included information such as age, educational level, marital status, job status, economic status, age at menarche, gravidity, history of abortion, and contraception methods used by the participants. Additionally, parts of the questionnaire developed in the UK were incorporated into the data collection process. These sections of the questionnaire focused on gathering information related to menstrual disorders, COVID-19 infection, and COVID-19 vaccination (12). Permission for translating the questionnaire was obtained from the original developers. Subsequently, 2 English-proficient reproductive health experts and a translator used the forward-backward method to translate the tool into Persian. The content validity of the translated tool was assessed by 10 experts comprising gynecologists, midwives, and reproductive health specialists. Additionally, 10 women of childbearing age evaluated the face validity of the questionnaire. The reliability of the questionnaire in the current study was assessed using the Cronbach's alpha coefficient (0.828). The survey's length, up to 56 questions, varied based on participants' COVID-19 infection and vaccination history and took about 20 min to complete.

The questionnaire utilized in this study lacked a comprehensive scoring system, necessitating the analysis and reporting of individual questions. In order to investigate factors linked to menstrual changes, participants were divided into 2 groups based on their experiences of menstrual alterations during the Covid-19 pandemic. Specifically, questions related to menstrual disorders, as outlined by the Figo criteria for abnormal uterine bleeding (questions 19, 20, 21, 25, 28, and 29), were consolidated. The Figo criteria encompass various factors such as menstrual cycle regularity, frequency, duration, volume, and intra-menstrual bleeding. Consequently, any affirmative response to these specified questions was construed as indicative of a change in menstrual patterns.

The acceptance of a change in menstruation post-vaccination relied on the individual's self-disclosure in question 41 of the questionnaire. Participants were asked to indicate whether they had noticed any changes to their menstrual cycles after receiving the COVID-19 vaccination. The response options included “No", “Yes, my menstrual cycles are more disrupted", “Yes, my menstrual cycles are less disrupted", and “I don't know".

In this study, the term “medication use history" specifically referred to the usage of non-steroidal anti-inflammatory drugs, antidepressants, and anti-anxiety drugs. Furthermore, the term “history of disease" encompassed an individual's medical background, including conditions such as endometriosis, thyroid disorders, uterine polyps, fibroids, polycystic ovaries, hyperprolactinemia, psychological illnesses, and chronic diseases like diabetes.

### Ethical considerations

The study was conducted in compliance with the ethical guidelines outlined in the 1964 Declaration of Helsinki. The research was approved by the Ethics Committee of Iran University of Medical Sciences, Tehran, Iran (Code: IR.IUMS.REC.1400.1173). Before participating in the study, written informed consent was obtained from all participants. Participants were also informed of their right to withdraw from the study at any point. It is worth noting that the questionnaire did not require participants' names.

### Statistical analysis

The statistical analysis was conducted using IBM SPSS (Statistical Package for the Social Sciences) Statistics software version 20 (IBM Corp., Armonk, NY, USA). The data were analyzed through descriptive statistics, including mean and standard deviation, as well as inferential statistics such as Chi-square and multivariate logistic regression. The significance level was set at p < 0.05.

## 3. Results 

Among the 1600 women invited in the study, a total of 1500 women (93.75%) agreed to participate and answer the survey questions. The mean age of the study subjects was 32.18 ± 7.07 yr. Approximately 40% of the participants had received an academic education, and 74% were married. The mean age at menarche was 12.74 ± 1.4 yr, and 32.8% of the women reported no history of pregnancy (Table I).

Before the onset of the COVID-19 pandemic, menstrual disorders were reported in 16.5% of women. The average menstrual cycle length before and after the pandemic was 26.83 ± 3.04 days and 26.93 ± 3.46 days, respectively, and the difference was not statistically significant (p = 0.106). Examining premenstrual symptoms, it was observed that before the pandemic, the average severity of premenstrual symptoms was 3.34 ± 2.41, while during the pandemic, it was 3.43 ± 2.49. The difference was found to be statistically significant (p = 0.002).

During the COVID-19 pandemic, 27.3% of the women reported changes in their menstrual duration, and 25.4% reported changes in their menstrual interval. Furthermore, 28.9% of the women experienced changes in their premenstrual symptoms, and 27% reported changes in their menstrual symptoms. The pattern of menstrual changes during the COVID-19 pandemic included a shorter interval in 8.6% of the participants, a longer interval in 8.9%, a shorter duration in 10.4%, a longer duration in 9.4%, heavier bleeding in 12.3%, and lighter bleeding in 14.7%. Additionally, participants had noticed changes in intra-menstrual bleeding compared to before the pandemic, with 13.4% reporting such alterations.

During the pandemic, 14.3% of women sought medical consultation due to changes in their menstrual cycle. With regards to medical history, a total of 320 women (21.3%) had a pre-existing history of gynecological, psychological, or chronic diseases. Among the participants, 60% reported having a prior infection with COVID-19.

Following the viral infection, 32.8% of the women experienced changes in the duration of their menstrual cycle, while 29.8% observed alterations in the interval between periods. Additionally, 34.5% noticed differences in the amount of menstrual bleeding. Specifically, 5.1% reported a shortened interval, whereas 5.4% experienced an extended interval.

Furthermore, 6.5% noted a decrease in the duration of their menstrual cycle, while 5.4% encountered an increase in duration. Moreover, 8.0% reported an increase in menstrual bleeding, and 7.1% reported a decrease in flow (Table II).

The menstrual cycle changes following vaccination are presented in table II. A significant majority of women (88.7%) had received COVID-19 vaccinations. Among these vaccinated women, the most commonly administered vaccine was Sinopharm (82.8%). Additionally, 18.1% of women reported changes in their menstrual cycles following vaccination (Table II).

To evaluate menstrual changes, participants who reported alterations in menstrual cycle regularity, frequency, duration, volume, and intra-menstrual bleeding during the COVID-19 pandemic were categorized into the menstrual changes group. The findings revealed that 52.6% of the women (n = 789) experienced menstrual changes during the pandemic. To explore the factors influencing these changes, a 2-by-2 comparison was conducted, which demonstrated significant relationships between menstrual changes and variables such as job status, number of pregnancies, contraception use, history of COVID-19 infection, and COVID-19 vaccination. The occurrence of menstrual changes was found to be associated with the dosage of the COVID-19 vaccine (p = 0.004). Specifically, 55.8% of women experienced changes in their menstrual cycles after the first dose, 51% after the second dose, and 56.8% after the third dose (Table III).

Subsequently, the significant variables were included in a logistic regression model using the forward method, revealing that both COVID-19 infection and vaccination against COVID-19 had statistical significance. Women infected with COVID-19 exhibited a 54% higher chance of experiencing menstrual changes compared to those who were not infected, and women who received three doses of COVID-19 vaccine had a 1.5 times higher chance of experiencing menstrual changes compared to unvaccinated women (Table IV).

**Table 1:** Frequency distribution of demographic and fertility-related characteristics (n = 1500)


**Demographic characteristics**		**Total (n = 1500)**
Age (yr)*		32.18 ± 7.07 (32, 10)
**< 30**		517 (34.5)
**30–39**		715 (47.7)
**≥ 40**		268 (17.9)
Education**		
	**Under diploma**	308 (20.5)
	**Diploma**	565 (37.7)
	**Academic**	627 (41.8)
Marital status**		
	**Single**	336 (22.4)
	**Married**	1110 (74.0)
	**Other**	54 (3.6)
Job status**		
	**Housekeeping**	925 (61.7)
	**Out of job**	93 (6.2)
	**Working at home**	66 (4.4)
	**Working outside home**	416 (27.7)
Economic status**		
	**Poor**	227 (15.1)
	**Moderate**	1143 (76.2)
	**Good**	130 (8.7)
**Age at menarche (yr)**		12.74 ± 1.40 (12, 2)
Gravida**		
	**0**	492 (32.8)
	**1**	277 (18.5)
	**2**	397 (26.5)
	**≥ 3**	334 (22.3)
Abortion**		
	**0**	732 (72.6)
	**1**	208 (20.6)
	**≥ 2**	68 (6.7)
Menstrual duration before the pandemic*		26.83 ± 3.04 (28, 3)
PMS severity before the pandemic*		3.34 ± 2.41 (3, 3)
Menstrual cycle length before the pandemic*		26.93 ± 3.46 (28, 2)
*Data presented as Mean ± SD (IQR, MD). **Data presented as n (%). PMS: Pre-menstrual syndrome		

**Table 2:** The change in menstrual cycle characteristics, COVID-19 infection and vaccination in women during the Covid-19 pandemic


**Variable**		**(n = 1500)**
**Change in menstrual cycle characteristics**		
	**Menstrual duration**	410 (27.3)
	**Menstrual interval**	381 (25.4)
	**PMS**	434 (28.9)
	**Pre-menstrual symptoms (abdominal pain, cramping, headache, breast tenderness, feelings of nervousness, frustration, fatigue, and alterations in libido)**	405 (27)
	**Menstrual symptoms (abdominal pain and cramping, irritability, despair, sensitivity, headache, clot expulsion, breast tenderness, fatigue, bloating, and gastrointestinal issues such as diarrhea)**	457 (30.5)
	**History of delayed period**	637 (42.5)
	**Intra-menstrual bleeding**	201 (13.4)
	**Amenorrhea**	95 (6.3)
**History of taking medications**		330 (22)
**Visiting doctor because of menstrual irregularities**		215 (14.3)
**Infection with COVID-19**		902 (60.1)
**Vaccination**		
	**1 dose**	95 (6.3)
	**2 doses**	635 (42.3)
	**3 doses**	600 (40.0)
**Vaccine type**		
	**Sinopharm**	1101 (82.8)
	**AstraZeneca**	109 (8.2)
	**Other**	71 (5.3)
	**Combination**	49 (3.7)
	**Not vaccinated**	170 (11.3)
**Menstrual changes following vaccination**		272 (18.1)
Data presented as n (%). PMS: Pre-menstrual syndrome, COVID-19: Coronavirus disease 2019		

**Table 3:** Factors associated with menstrual changes (n = 1500)


**Factors**		**No menstrual changes (n = 711)**	**Menstrual changes (n = 789)**	**P-value**
**Age (yr)**				
	**< 30**	244 (34.3)	273 (34.6)	
	**30–39**	356 (50.1)	359 (45.5)	
	**≥ 40**	111 (15.6)	157 (19.9)	0.064
**Job status**				
	**Housekeeping**	480 (51.9)	445 (48.1)		
	**Out of job**	44 (47.3)	49 (52.7)	
	**Working at home**	19 (28.8)	47 (71.2)	
	**Working outside home**	168 (40.4)	248 (59.6)	0.000
**Gravidity**				
	**0**	198 (40.2)	294 (59.8)		
	**1**	131 (47.3)	146 (52.7)		
	**2**	211 (53.1)	186 (46.9)	
	**≥ 3**	171 (51.2)	163 (48.8)	0.001
**Contraception use**				
	**None**	167 (42.2)	229 (57.8)		
	**Hormonal**	149 (40.9)	215 (59.1)		
	**None-hormonal**	395 (53.4)	345 (46.6)	
**Infection with COVID-19**		365 (40.5)	537 (59.5)	0.000
**Received dose of COVID-19 vaccination**				
	**1 dose**	42 (44.2)	53 (55.8)		
	**2 doses**	311 (49)	324 (51)		
	**3 doses**	259 (43.2)	341 (56.8)	0.004
Chi-Square test, COVID-19: Coronavirus disease 2019				

**Table 4:** Logistic regression analysis of factors associated with menstrual changes


**Variables**		**B**	**P-value**	**Exp (B)**	**95% CI for Exp (B)**
**Job (Ref: Working outside home)**					
	**Housekeeping**	-0.222	0.124	0.801	0.603–1.063
	**Out of job**	-0.355	0.136	0.701	0.440–1.118
	**Working at home**	0.504	0.089	1.655	0.926–2.957
**Gravidity (Ref: ≥ 3)**					
	**0**	0.300	0.129	1.350	0.916–1.989
	**1**	0.139	0.409	1.149	0.826–1.597
	**2**	-0.089	0.562	0.915	0.678–1.235
**Contraception use (Ref: no)**					
	**Hormonal**	0.303	0.109	1.353	0.935–1.959
	**Non-hormonal**	-0.100	0.579	0.905	0.636–1.287
**Infection with COVID-19 (Ref: yes)**					
	**No**	-0.608	0.000	0.545	0.438–0.678
**COVID-19 vaccination (Ref: no)**					
	**1 dose**	0.428	0.109	1.534	0.909–2.589
	**2 doses**	0.207	0.256	1.231	0.860–1.760
	**3 doses**	0.388	0.034	1.474	1.029–2.113
CI: Confidence interval, Ref: Reference, Exp: Exponential value, COVID-19: Coronavirus disease 2019					

## 4. Discussion

The study examined menstrual cycle changes during the COVID-19 pandemic among Iranian women of reproductive age. Findings revealed that 52.6% of women who had menstruated in the previous year reported alterations in their menstrual patterns. Around one-third noticed changes in cycle duration, interval, and premenstrual symptoms. Specific changes included 8.6% with shorter intervals, 8.9% with longer intervals, 10.4% with reduced flow duration, and 9.4% with increased flow duration. Additionally, 12.3% experienced heavier blood loss, while 14.7% had lighter blood flow.

In a systematic review and meta-analysis conducted in Iran on the prevalence of menstrual disorder, 13.11% of Iranian women were shown to have oligomenorrhea, 9.94% polymenorrhoea, 12.94% hypermenorrhea, 5.25% hypomenorrhea, 19.24% menorrhagia, and 6.04% metrorrhagia (17). Similar to our results, a study in Spain reported that over one-third of the women experienced menstrual changes during the COVID-19 pandemic (9). Furthermore, a study from Turkey revealed women of childbearing age experienced a decrease in menstrual cycle length and amount of menstrual bleeding during the pandemic compared to the period before it (7). The menstrual cycle is regulated by a complex interplay of endocrine, autocrine, and paracrine factors, leading to various causes of menstrual disturbances (18, 19). Psychological stress, a recognized factor for hypothalamic hypogonadism, can impact menstrual frequency, the amount of menstrual bleeding, and the duration of menstruation (15). Given the impact of stress on the hypothalamic-pituitary-gonadal axis, high levels of stress experienced over the COVID-19 pandemic may alter menstrual cycle patterns (6). COVID-19 mitigation strategies, such as lockdowns and social distancing, have increased stress, depression, and anxiety levels among women, potentially contributing to menstrual changes (6, 9, 15, 19). Studies have suggested that COVID-19-related stressors play a role in menstrual irregularities (6, 8). However, a study from Great Britain and the United States showed that the pandemic did not induce widespread changes to menstruation among women, with fewer women experiencing menstrual abnormalities during the pandemic than before. The researchers proposed that improved health habits, such as increased exercise and better sleep patterns during the pandemic, may have contributed to the reduction in menstrual abnormalities (5).

The study found that a considerable number of women who had previously infected with COVID-19 reported changes in their menstrual cycles, such as variations in interval, duration, and flow volume. Infection with COVID-19 linked to a 54% higher chance of experiencing menstrual alterations. This corresponds with findings from a study in Jordan and Iraq, where 47.2% of women with COVID-19 showed changes in the duration between periods and blood loss volume (19). Psychological stress is widely acknowledged as a long-term symptom of COVID-19 infection (20). In attempts to elucidate the underlying mechanisms linking COVID-19 infection and menstrual changes, some researchers have postulated that systemic inflammation caused by COVID-19 may reduce the circulation of 25 hydroxyvitamin D (21, 22).

Vitamin D deficiency has implicated in menstrual irregularities and pre-menstrual syndrome. Additionally, COVID-19 infection can deplete vitamin B6 levels, thereby increasing estrogen levels and resulting in heavy and painful menstruation (19). Alternatively, specific interactions between the reproductive system and COVID-19 infection at the ovarian and endometrial levels could exist. It is plausible that COVID-19 infection may influence the production of ovarian hormones and/or the endometrial response during menstruation. For instance, altered numbers of endometrial leukocytes during COVID-19 infection may affect menstrual blood loss. COVID-19 has also been related to endothelial cell dysfunction and changes in the coagulation system, which are both vital components of endometrial function during menstruation. These observations provide a potential mechanism through which COVID-19 could disrupt menstrual patterns (15). Moreover, a study has demonstrated a link between infection-induced immune system disruption and the exacerbation of premenstrual symptoms (12).

In our study nulligravida women, employed individuals, and those using hormonal contraception were more prone to experiencing menstrual changes during the pandemic. However, apart from a history of COVID-19 infection and vaccination, other variables did not show a significant link to menstrual changes. Our findings revealed that 18.1% of women noticed alterations in their menstrual cycles post-COVID-19 vaccination. Specifically, women who received 3 doses of a COVID-19 vaccine had 1.5 times higher chances of experiencing menstrual changes compared to the unvaccinated, regardless of the vaccine type. Previous research has also highlighted menstrual changes associated with mRNA and adenovirus vectored COVID-19 vaccines (11, 15). Additional studies (14, 23, 24) have supported the link between menstrual cycle length changes and COVID-19 vaccines. Women who received 2 doses of a COVID-19 vaccine within a menstrual cycle experienced an increase in cycle length of 3.7 days compared to those who were not vaccinated (23). Similarly, a study conducted on 4942 women from 6 Arab countries in the Middle East region showed that fully vaccinated women against COVID-19 reported heavier menstrual flow and more days of bleeding (24). Another study highlighted that approximately 60% of childbearing-age women reported menstrual irregularities following the first dose of any COVID-19 vaccine, regardless of the vaccine type (14). The underlying mechanism appears to be related to the immune response triggered by the vaccines rather than a specific vaccine component (11). The activation of the immune system by the vaccines may lead to the attack of immune cells and inflammatory molecules in the uterus, potentially resulting in menstrual cycle changes (25). Furthermore, the diffusion of the spike protein in women's tissues, whether related to COVID-19 infection or released following mRNA vaccination, has the potential to disrupt the endocrine homeostasis of the menstrual cycle (14). However, the exact mechanism by which vaccines affect the menstrual cycle remains undetermined (15), and further investigation through longitudinal studies is necessary.

Our study has limitations that should be considered when interpreting the results. The cross-sectional design used does not allow for definitive causal relationships to be established between the COVID-19 pandemic and menstrual changes. Additionally, relying on self-reported data, including details on COVID-19 diagnosis, gynecological issues, and other medical conditions, may introduce recall bias and should be considered.

## 5. Conclusion

Our study uncovered a substantial prevalence of menstrual changes among Iranian women of childbearing age during the COVID-19 pandemic. We found a significant association between experiencing menstrual changes and a history of COVID-19 infection and COVID-19 vaccination. Although our study did not seek to establish a causal relationship between COVID-19 vaccination and menstrual cycles, there were reports of menstrual changes following vaccination (26).

Healthcare professionals should be knowledgeable about the menstrual changes associated with COVID-19 infection and vaccination, to provide information and support to affected women. Our findings underscore the urgent need for further investigation into the long-term reproductive consequences of the COVID-19 pandemic. Such research can enhance our understanding of how external factors influence the menstrual cycle and ultimately contribute to improving women's menstrual and reproductive health. Examining menstrual health, in the context of COVID-19, presents an opportunity to inform policies and practices that address gender inequalities in healthcare and society. By leveraging these insights, we can effectively “build back better" in a post-COVID-19 world.

##  Data availability

The datasets used and analyzed in the current study can be provided by the corresponding author upon a reasonable request.

##  Author contributions

Fahimeh Ranjbar and Maryam Gharacheh played pivotal roles in the study's conceptualization, project supervision, and provision of crucial insights during the interpretation of study results and manuscript drafting. Shima Haghani conducted the data analysis, while Motahareh Aghashahi was responsible for data collection and entry. Furthermore, Shima Haghani and Motahareh Aghashahi actively participated in the article review process. All authors approved the final manuscript and take responsibility for the integrity of the data.

##  Conflict of Interest

The authors declare that there is no conflict of interest.
